# Antimicrobial, Antioxidant, and Immunomodulatory Properties of Essential Oils: A Systematic Review

**DOI:** 10.3390/nu11112786

**Published:** 2019-11-15

**Authors:** Magdalena Valdivieso-Ugarte, Carolina Gomez-Llorente, Julio Plaza-Díaz, Ángel Gil

**Affiliations:** 1 Institute of Nutrition and Food Technology “José Mataix”, Center of Biomedical Research, University of Granada, Avda. del Conocimiento s/n. 18016 Armilla, Granada, Spain; malenavaldivieso@gmail.com (M.V.-U.); jrplaza@ugr.es (J.P.-D.); agil@ugr.es (Á.G.); 2 Department of Biochemistry and Molecular Biology II, School of Pharmacy, University of Granada, 18071 Granada, Spain; 3 ibs.GRANADA, Instituto de Investigación Biosanitaria, Complejo Hospitalario Universitario de Granada, 18014 Granada, Spain; 4 CIBEROBN (CIBER Physiopathology of Obesity and Nutrition), Instituto de Salud Carlos III, 28029 Madrid, Spain

**Keywords:** essential oils, volatile oils, antimicrobial, antioxidant, immunomodulatory, food preservation, biofilm

## Abstract

Essential oils (EOs) are a mixture of natural, volatile, and aromatic compounds obtained from plants. In recent years, several studies have shown that some of their benefits can be attributed to their antimicrobial, antioxidant, anti-inflammatory, and also immunomodulatory properties. Therefore, EOs have been proposed as a natural alternative to antibiotics or for use in combination with antibiotics against multidrug-resistant bacteria in animal feed and food preservation. Most of the results come from in vitro and in vivo studies; however, very little is known about their use in clinical studies. A systematic and comprehensive literature search was conducted in PubMed, Embase^®^, and Scopus from December 2014 to April 2019 using different combinations of the following keywords: essential oils, volatile oils, antimicrobial, antioxidant, immunomodulation, and microbiota. Some EOs have demonstrated their efficacy against several foodborne pathogens in vitro and model food systems; namely, the inhibition of *S. aureus*, *V. cholerae*, and *C. albicans* has been observed. EOs have shown remarkable antioxidant activities when used at a dose range of 0.01 to 10 mg/mL in cell models, which can be attributed to their richness in phenolic compounds. Moreover, selected EOs exhibit immunomodulatory activities that have been mainly attributed to their ability to modify the secretion of cytokines.

## 1. Introduction

Foodborne-related diseases are an increasingly major public health problem worldwide [1]. Microbial contamination is one of the factors in developing foodborne diseases and food spoilage [2]. Since ancient times, different methods to preserve food for a longer period have been sought. For this reason, and thanks to the advancement of knowledge and the rapid development of new technologies, different chemical compounds have been developed, commonly known as additives, which extend the life of foods or are used as sweeteners or coloring agents [3]. However, the presence of pathogenic microorganisms continues to result in large economic losses and multiple diseases in humans [4]. On the other hand, the indiscriminate use of antibiotics in both humans and animals against pathogenic microorganisms has contributed to the extension of resistant and even multidrug-resistant bacterial strains [4,5]. In recent years, a tendency to use natural additives, mainly due to the increasing desire for the consumption of minimally processed products, has emerged [4,6]. Therefore, there is a need for alternative natural compounds that can perform the same function of common additives or can be used as an alternative to antibiotics. One such possibility is the use of essential oils (EOs) due to their known antimicrobial, antioxidant, immunomodulatory, and food preservative activities [7]. In line with this, several studies have described the antibacterial activity of EOs, underlying their effective use on multidrug-resistant strains [8,9].

EOs, also known as “volatile oils”, are complex mixtures of volatile compounds that are produced by aromatic plants as secondary metabolites. They are responsible for the aromatic plant’s properties, and for this reason, they are characterized by their strong smells [10]. In general, EOs are liquid, volatile, and soluble in lipids and organic solvents. They can be present in all plant organs, including buds, flowers, leaves, seeds, stems, flowers, fruits, roots, wood, or bark. Different extraction techniques are widely employed for the extraction of EOs such as steam distillation, solvent extraction, and supercritical fluid extraction [11,12]. These EOs are characterized by the presence of variable mixtures of bioactive compounds, mainly terpenoids, especially monoterpenes and sesquiterpenes. Some of them also contain nonterpenic compounds biogenerated by the phenylpropanoid pathway, such as eugenol, cinnamaldehyde, and safrole [13]. These bioactive compounds are responsible for the biological properties of EOs. Among them, terpenoids are the bioactive compounds that have a more important role in pathogen resistance [14]. Specifically, monoterpenoids affect the multiplication and development of microorganisms by interfering with their physiological and biochemical processes during their development and multiplication [15]. Cinnamon bark oil is one of the most effective EOs against common foodborne pathogens [16]. It should be noted that the effect of EOs on bacterial growth will depend on whether they are Gram-positive or Gram-negative bacteria, since the lipopolysaccharide (LPS) layer in Gram-negative bacteria acts as a barrier for macromolecules and hydrophobic compounds such as those present in EOs [15]. Using EOs to extend the shelf life of fish and meat has also been reported in previous studies. Examples include the preservative effect of lemon EOs on salted sardines [17], the effect of chitosan coatings enriched with cinnamon oil on the quality of rainbow trout (*Oncorhynchus mykiss*) during refrigerated storage [18], and the lengthening of the storage period of red sea bass by means of clove, cumin, and peppermint oils or poultry meat in thyme oil [19].

The antioxidant activity of EOs is another biological property of great interest because they may preserve foods from the toxic effects of oxidants [20]. It is noteworthy to mention that EOs have also been shown to possess a wide range of immunomodulatory properties. To date, a few studies dealing with the immunomodulatory effect of EOs have been reported [21,22,23]. In this regard, monoterpenes have been shown to exert a strong immunobiological effect through their effect on tumor necrosis factor (TNF)-α, interleukins (ILs), thromboxane, and leukotriene production [24]. This immunomodulatory activity indicated the possibility of using EOs as ingredients in functional foods. 

Most of the results of the antimicrobial, antioxidant, and immunomodulatory effects of EOs come from in vitro and in vivo studies. However, very little is known about their use in clinical studies. The present work aims to perform a systematic review of the scientific literature on the important biological properties of EOs in food preservation and to describe the antimicrobial, antioxidant, and immunomodulatory properties that render them desirable for use in functional foods. 

## 2. Materials and Methods

### 2.1. Search Strategy

In this review, the specialized databases PubMed (US National Library of Medicine National Institutes of Health), Embase^®^, and Scopus were used for the literature search from December 2014 to April 2019, with the aim of restricting the search to the articles that make use of the most modern techniques, using different combinations of the following keywords: essential oils, volatile oils, antimicrobial, antioxidant, immunomodulation, and microbiota. In PubMed, we used the following search equation strategy: (“essential oils” [All Fields] OR “volatile oils” [All Fields]) AND (“immunomodulation” [All Fields] OR “drug resistance, multiple, bacterial” [All Fields] OR “immune system” [All Fields]). When we used PubMed, we included Medical Subject Heading (MeSH) terms to increase the power of the search. The search equation used in Scopus was: “essential AND oils” AND immunomodulation AND drug resistance, multiple, bacterial. 

### 2.2. Selection Criteria 

Articles were organized by the antimicrobial and immunomodulatory effects of EOs; after that, two members of the team (M.V.-U. and C.G.-Ll.) extracted information about the characteristics of the studies. The information extracted from the articles included EOs, cell lines (in vitro studies), animal models (in vivo studies), doses or concentrations, routes of administration, biochemical assays, and the molecular mechanisms investigated. The quality assessment and selection were performed by two authors (M.V.-U. and C.G.-Ll.) who independently worked according to the main criteria of PICO (Population, Intervention, Comparison, Outcome) (Table 1); in case of discrepancies, a third independent reviewer (J.P.-D.) was consulted for the final decision.

### 2.3. Data Handling, Analyses, and Extraction

The inclusion criteria used were the following: (1) studies with EOs with antimicrobial and immunomodulatory properties in vitro and in vivo; (2) food and nutrition-related studies; and (3) studies with significant results obtained through appropriate statistical analysis. The exclusion criteria used were as follows: (1) studies written in languages other than English or Spanish; (2) the use of plant extracts or derivatives, instead of EOs; (3) review articles, conference proceedings, and editorials/letters; (4) studies without controls; and (5) agar diffusion method as the only assay for the study of the antimicrobial effect. After removing duplicates, acceptability for inclusion was evaluated based on the following: (1) reading the title and abstract; and (2) reading the full text. 

We provided a narrative synthesis of the main results of the selected articles. These results were classified according to the EOs’ properties: (i) antimicrobial activity, (ii) antioxidant activity, and (iii) immunomodulatory effects of EOs in cells and animals. 

## 3. Results

### 3.1. Study Identification and Selection

**Figure 1:**
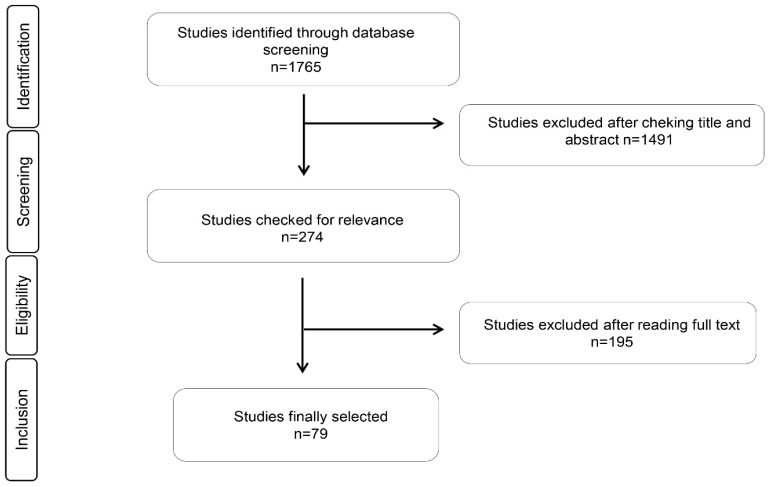
PRISMA flow chart for studies related with antibacterial, antioxidant, and immunomodulatory properties of essential oils.

Seventy-nine relevant articles were identified, which was in agreement with our inclusion and exclusion criteria. The selected articles were grouped into the antimicrobial and antioxidant effects of the EOs, immunomodulatory effects of EOs in cell studies, and animal supplementation with EOs. We only found one article in human samples that met our eligibility criteria. We did not find any intervention studies in humans. The complete process is explained in Figure 1, which is based on a PRISMA flow chart.

### 3.2. Antimicrobial Activity of Esential Oils

Table 2 lists the selected publications and their main results of the antimicrobial effect of EOs. In total, 49 articles were identified. Forty-three articles described the effects of EOs over several bacterial strains such as *E. coli*, *S. aureus*, *B. cereus*, and *P. aeruginosa*. In six articles, the inhibition of biofilm formation by EOs was described. The dose used for the determination of minimum inhibitory concentration (MIC)/minimum bactericidal concentration (MBC) values ranged from 9 to 229 mg/mL, and the incubation time varied from 18 to 24 h.

### 3.3. Antioxidant Activity of Essential Oils

Table 3 depicts the articles and their main results regarding the antioxidant effects of EOs. Twenty-one studies were selected. The main methods used to evaluate the antioxidant capacity were the following: measuring 1,1-diphenyl-2-picrylhydrazyl (DPPH) and 2′-azino-bis(3-ethylbenzothiazoline-6-sulphonic acid) (ABTS) radicals scavenging activity, ferric-reducing antioxidant power (FRAP), and oxidative DNA damage protective effect induced by Fe^2+^ and 2,2′-azobis (2-methylpropionamidine) dihydrochloride (AAPH). EOs demonstrated ability in antioxidant assays in a concentration-dependent manner. The dose range was 0.05% to 3.2% *v*/*w*, 0.1 to 10 mg/mL, and 12.5 to 2000 µg/mL.

### 3.4. Immunomodulatory Activity of Essential Oils in Cells and Animals 

Table 4 and Table 5 describe the main effects of EOs in cellular and animal studies, respectively. Eight of the selected studies were specifically conducted in cells, whereas five out of 14 publications selected performed antimicrobial studies against different bacterial strains and immunomodulatory studies in a cellular model. Most of the studies were conducted in animal cells (nine out of 13), although studies in human cells were also performed (seven out of 14). The dose ranged from 1.25 to 1000 μg/mL, and the incubation times varied from 1 to 72 h. In the case of animal studies, six out of 10 were performed on poultry animals, while three out of 10 were performed on C57BL/6 mice. One article was performed in silver catfish. The dose administrated varied from 7.5 mg/kg to 1 kg/ton in weaned piglets. 

## 4. Discussion

There is a huge amount of different EOs from different plants around the world. Most of them have been shown to exert a well-characterized antimicrobial activity against Gram-positive and Gram-negative bacteria, but also against other microorganisms, such as yeast. The irreversible damage of the bacterial cell wall and membrane has been proposed as its main mechanisms of action. In addition, several studies revealed how EOs can inhibit biofilm formation through the inhibition of bacterial cell communication. Regarding their antioxidant and immunomodulatory properties, EOs have been shown to exert a protective effect through their radicals scavenging activity, with an inhibition percentage range of 20%–70%, and their effect against DNA oxidative damage induced by Fe^2+^. Regarding their immunomodulatory effect, EOs or their main compounds can modulate the secretion of important cytokines in a cell culture challenge with LPS. This capacity was evident in their effect in inflammatory pathways such as nuclear factor kappa-light-chain-enhancer of activated B cells (NF-κB). It is important to highlight that a cytotoxic effect was not observed when EOs where used at low concentrations. There have been a few studies in poultry animals where EOs have a positive effect on growth parameters; however, on the gastrointestinal microbiota, EOs have a negative affect on gastrointestinal pathogen microorganisms. Their biological properties can be attributed to their complex composition with more than 300 different volatile compounds. These volatile compounds include terpenes, alcohols, phenolic acids, ethers, esters, amines, amides, ketones, and aldehydes, among other chemical components [102]. Although most of their biological actions have been related to their main components, it is important to underpin that the aforementioned properties come over the synergic effect of all the components. The results from this review indicate that EOs have important biological properties that make them suitable for use in the development of functional foods. However, in this regard, one aspect that must be considered is their strong smell, which could result in low acceptance by the consumer or modification of the organoleptic properties of the food [8].

### 4.1. Antimicrobial Activity of Esential Oils

In recent years, there has been a growing interest in researching and developing new antimicrobial agents from EOs due to drug resistance in foodborne bacterial enteric pathogens. Numerous publications have presented data on the antimicrobial properties of EOs [29,30]. 

A variety of laboratory methods can be used to evaluate the in vitro antimicrobial activity of an EO. The most well-known and basic methods are the disk diffusion and broth or agar dilution methods [103]. The lowest concentration of antimicrobial agent that completely inhibits the growth of the organism is called the minimum inhibitory concentration (MIC). The most appropriate assays for the determination of the MIC value are the dilution methods, as they offer the possibility of a precise estimation of the concentration of the tested antimicrobial agent.

The antibacterial effects based on the MIC determination of several EOs alone or in combination against different food-associated Gram-positive and Gram-negative bacteria have been described. Parsley, lovage, basil, and thyme are a few of the aromatic herbs commonly used in industry with low-cost production. Different parts of these herbs (leaves, flowers, stems, fruits, and seeds) have been used to extract EOs [60]. Parsley and lovage EOs revealed no inhibitory effects against all tested strains. Thyme EO had the highest percentage yield and antibacterial potential from all tested formulations; its use in combination with parsley, lovage, and basil EOs results in a reduction in its antibacterial activity; therefore, thyme EO should be used alone [60]. EOs of cultivated oregano (*Origanum vulgare*), sage (*Salvia officinalis*), and thyme (*Thymus vulgaris*) have been shown to exert a potent antimicrobial effect. Among them, the most efficient were the EOs from thyme, followed by those of oregano. With MIC values above 150 mg/mL, sage EOs did not show any antibacterial effect against the majority of the bacterial strains [38]. Three *Origanum* species analyzed, *O. dictamnus* and *O. microphyllum*—both endemic in Greece—and *O. libanoticum*, endemic in Lebanon, were evaluated, but only *O. dictamnus* exerted antibacterial activity [46].

Different bacterial and fungal strains have been used to determine the antibacterial effects of different Eos; these microorganisms comprise strains from *Staphylococcus*, *Bacillus*, *Listeria*, *Helicobacter*, *Micrococcus*, *Pseudomonas*, *Klepsiella*, *Escherichia*, *Salmonella*, *Enterobacter*, and *Candida*. EOs from *Heracleum pyrenaicum* subsp. *orsinii*, *Pistacia vera* L., *Myrcia ovata Cambessedes*, *Thymus bovei*, *Minthostachys verticillata*, *Allium roseum*, *Petroselinum crispum*, *Satureja bachtiarica* Bunge, *Ocimum suave*, *Jatropha gossypifolia* L., and *Juniperus rigida* have been shown to exert antibacterial and anti-yeast effects [34,37,41,43,47,48,51,64,67,68,69]. One of the proposed mechanisms for those effects is the irreversible damage of the bacteria cell wall and membrane, which leads to not only a leakage of proteins but also of DNA and RNA molecules [47,48].

*Enteromorpha linza*, *Baccharis dracunculifolia*, *Syringa yunnanensis*, *Senecio nutans*, basil, chamomile blue, oregane, thyme, tea tree oil, *Carum copticum*, and *Xanthium strumarium* L. EOs have also been described for their anti-microorganism effects against several bacteria, fungi, and even some pathogens, such as *Vibrio cholerae*. Specifically, *Enteromorpha linza* EO is effective against *B. cereus* and *S. aureus* [54], *Baccharis dracunculifolia* EO is active against *S. aureus* and *E. coli* [55], *Senecio nutans* EO is effective against *V. cholerae* [53], *Syringa yunnanensis* EO is effective against *S. aureus* [72], *Carum copticum* EO is capable of reducing the growth of *E. coli* O157:H7 [44], and *Xanthium strumarium* L. EO is also effective against *S. aureus*, *B. subtilis*, *K. pneumoniae*, *P. aeruginosa*, *C. albicans*, and *A. niger* [63]. In contrast, basil, chamomile blue, oregane, thyme, and tea tree oil EOs were not sufficiently effective against *A. baumannii*, *E. coli*, *K. pneumoniae*, and *P. aeruginosa* [58]. 

EOs from plants from different regions of the world have been studied. In this sense, EOs derived from *Aloysia citriodora Palau*, which is harvested in different regions of Morocco, showed significant antimicrobial activity against both Gram-negative and Gram-positive bacteria [52]. The EOs of *Peperomia pellucida*, an herbaceous plant from the Amazon region, exhibited strong antibacterial activities against six different bacteria strains [50]. Salem et al. evaluate the biological activity of the EOs derived from *Corymbia citriodora* leaves and *Cupressus macrocarpa* from Egypt. While the antibacterial activity of EO from *C. citriodora* leaves has MIC values ranging from 0.06 to 0.20 mg/mL, EO from *C. macrocarpa* branchlets showed less activity against bacterial strains [59]. 

In recent years, there has been a dramatic increase in resistance to antimicrobial drugs against *Salmonella* Enterica and *Campylobacter* spp. *Campylobacter* spp. is one of the most common causative agents of gastroenteritis in the world, whereas salmonellosis is a major foodborne disease worldwide. Bacteria can be transmitted to humans by the consumption of contaminated poultry, eggs, beef, milk, juices, fruits, and vegetables. Several studies have shown that EOs could be used as alternative therapeutics to treat antibiotic-resistant *Salmonella*. In this regard, *Ruilopezia bracteosa* EO has been described as being effective against *S. aureus* and *E. faecalis* compared with several antibiotics [26]. Similarly, Ashraf et al. studied the effect of *Nigella sativa* (Black seed) oil against antibiotic-resistant isolates by a well diffusion and microbroth dilution method, and they concluded that *N. sativa* had in vitro activity against *Salmonella* Enterica [27]. Chiboub et al. evaluated the biological activity of the EOs of two varieties of *Foeniculum vulgare* in the growth of *Salmonella* Enterica, and the results showed a significant antimicrobial activity [32]. Aghraz et al. showed that EOs from *Cladanthus arabicus* and *Bubonium imbricatum* contain a potent activity against the tested *Salmonella strain*, with MIC values between 200 and 800 μg/mL for *C. arabicus* and from 400 to 1600 μg/mL for *B. imbricatum* [25]. The evaluation of the synergistic effect of mixed EOs was also investigated. To increase the sensitivity against the *Salmonella* Typhimurium strain, a mixture of *Thymus vulgaris* L., *Rosmarinus officinalis* L., and *Myrtus communis* L was used. EOs were used in combined treatment using an experimental design methodology [36]. A mixture of 55% of *T. vulgaris* L. and 45% of *M. communis* L. EOs, respectively, can be considered for the increase of *Salmonella* Typhimurium sensitivity. Mutlu-Ingok et al. studied the antibacterial activities of cardamom, cumin, and dill weed EOs against *Campylobacter jejuni* and *Campylobacter coli*. The results indicated that EOs might be effective inhibitors by directly acting at the bacterial membrane integrity level [49]. It is important to highlight that EOs derived from oregano, thyme, clove, and arborvitae also showed a very strong antibacterial activity against other bacteria causing foodborne disease; therefore, they can be used as antimicrobial agents [57].

One important concern in the food industry is the presence of biofilms. Bacteria can be suspended in liquid food, usually living planktonically, although they can easily adhere to the surface of food materials and food processing equipment, forming a bacterial biofilm. Biofilms are microbial communities that are characterized by their adhesion to solid surfaces and the production of a matrix of exopolymeric substances; the matrix consists of polysaccharides, proteins, DNA, and lipids, which surround the microorganisms, proffering structural integrity and a unique biochemical profile to the biofilm [104]. Biofilms can exist on all types of surfaces in food plants ranging from plastic, glass, metal, and wood, to food products [105], resulting in food spoilage and economic losses for the producers [105]. Several studies revealed how EOs can inhibit biofilm formation [33,56,62,65,71]. *Cinnamomum zeylanicum* oil may be a useful approach to impair the biofilm produced by Gram-negative bacteria [33]. According to Porfirio et al., *Lippia Alba* EOs have a strong inhibition of *S. aureus* biofilm formation [56]. Likewise, EOs derived from parsley and basilic can inhibit and eradicate the mature biofilm formed by *Vibrio* strains on a polystyrene surface even at low concentrations. These two EOs could be used to prevent and eradicate the contamination of sea products by these strains [65]. It has been described that quorum sensing (QS), the process through which bacterial cells communicate with each other by releasing, sensing, and responding to small diffusible signal molecules [106], is involved in biofilm formation. QS has been inhibited by the EOs of several plants, such as *Thymus daenensis* and *Satureja hortensis*. Consequently, EOs act as anti-biofilm and QS inhibitor agents against bacteria [62].

### 4.2. Antioxidant Activity of Essential Oils

The excessive amounts of reactive oxygen species (ROS) can lead to the peroxidation of lipids, glycation/oxidation/nitration of proteins, inactivation of enzymes, DNA damage, and other alterations in the cellular organelles [107,108].

In recent years, food oxidation and food spoilage caused by microorganisms form one of the most important issues facing the food industry and consumers. Accompanied by growing consumer interest in natural food additives, the search for effective antioxidants and antibacterial agents from natural resources as alternatives to suppress food deterioration is now focused on edible plants, since they present with fewer side effects than the synthetic chemicals used in today’s foods [109]. There has been an increasing realization in recent years that several plant-derived EOs may possess antioxidant, antimicrobial, anticancer, and apoptosis-inducing properties [110].

*Cyperus rotundus* L. is a smooth and perennial weed that is widely distributed in tropical and warmer temperate regions worldwide [77]. The antioxidant properties of the *C. rotundus* rhizome were determined. In addition, 1,1-diphenyl-2-picrylhydrazyl (DPPH) and 2,2′-azino-bis(3-ethylbenzothiazoline-6-sulphonic acid) (ABTS) radicals scavenging activity, ferric-reducing antioxidant power (FRAP), and oxidative DNA damage protective effect induced by Fe^2+^ and 2,2′ -azobis (2-methylpropionamidine) dihydrochloride (AAPH) were also determined. *C. rotundus* rhizomes possessed an excellent antioxidant activity, as evidenced by in vitro DPPH, ABTS, and FRAP assays. In addition, EOs exhibited a protective effect against DNA oxidative damage induced by Fe^2+^ and AAPH, respectively [77].

An antioxidant combination effect of bay leaf, black pepper, coriander (seed and leaf), cumin, garlic, ginger, mustard, onion, and turmeric EOs was assessed by the DPPH free radical scavenging method. Only the coriander/cumin seed oil combination exhibited antioxidant activity in a synergistic interaction. Bioactive compounds responsible for this antioxidant capacity were linalool from coriander seed oil and p-coumaric acid from cumin seed oil [74]. DPPH radical scavenging activity assay, β-carotene bleaching test (BCBT), and ABTS assay were determined in *Melissa officinalis* and *Dracocephalum moldavica* EOs. Both EOs showed a strong activity in terms of the maintenance of β-carotene molecules. The ABTS radical scavenging of the EOs was dose-dependent and increased with the increase in the EOs concentration [76]. The antioxidant activity of the EO of *Ruta chalepensis* was tested by DPPH using Trolox as a reference compound. Percentages of inhibition for *R. chalepensis* collected from Jerusalem, Hebron, and Jenin were 69.56%, 61.53%, and 24.12%, respectively [78]. *Achillea millefolium* L., *Anethum graveolens* L., and *Carum copticum* L. EOs were selected to evaluate their antioxidant properties using DPPH, FRAP, BCBT, and total phenolic content assays. *A. millefolium* EO had the highest antioxidant activity in all conducted assays [79]. With a similar methodology, *Foeniculum vulgare*, *Petroselium crispum*, and *Lavandula officinalis* EOs, six different populations of *Origanum heracleoticum* L. from Calabria (Italy) Eos, and *Pelargonium asperum* and *Ormenis mixta* were analyzed. *Petroselium crispum* had the highest phenolic content and the best antioxidant profile [80], EO samples from Bagaladi and Longobucco were the most active in DPPH and BCBT assays [81], and only *Ormenis mixta* EO displayed an effective antioxidant ability, as tested by DPPH assay [83].

The antioxidant properties of EOs from the fruits *Dennettia tripetala* G. Baker as ripe and unripe fruit oil were tested. The ripe fruit EO has shown higher antioxidant strength than unripe fruit EO and vitamin C, but a lower activity compared to BCBT. The EOs also demonstrated strong ability in terms of scavenging three other different radicals (ABTS, lipid peroxide, and nitric oxide radicals) in a concentration-dependent manner [82]. With a similar methodology, *Jatropha gossypifolia* L and *Peperomia pellucida* (L.) Kunth were tested. The EOs effectively reduced oxidants to neutral molecules in a concentration-dependent manner [50,51].

*Ferulago angulata*—collected from natural habitats in the alpine regions of southwestern Iran—balsam fir (*Abies balsamea* (L.) Mill.), black spruce (*Picea mariana* (Mill.) B.S.P.), white spruce (*Picea glauca (Moench) Voss*), tamarack (*Larix laricina (Du Roi) K. Koch)*, jack pine (*Pinus banksiana Lamb.*), eastern white cedar (*Thuja occidentalis L.*), Labrador tea *(Ledum groenlandicum L.)*, *Mentha spicata* EOs, and the EO of the *Pistacia vera* L. variety Bronte were analyzed using DPPH assay. The highest antioxidant activity was obtained from the EO of the Kallar population [84]; in contrast, balsam fir, black spruce, white spruce, tamarack, and eastern white cedar oils again exhibited very poor antioxidant activities [85]. The antioxidant ability of the spearmint oil was 3 μg/mL, in comparison to 11.5 μg/mL for the standard compound. This interesting biological activity can be explained by the presence of the monoterpenes limonene, terpinolene, γ-terpinene, 1,8-cineole, and carvone in the EO [88]; the *Pistacia vera* L. variety Bronte showed little affect against the DPPH test [64]. 

The antioxidant properties of aerial parts of *Glycyrrhiza triphylla* Fisch. and CA Mey and parsley, lovage, basil, and thyme EOs were investigated with DPPH and BCBT assays. *G. triphylla* EO exhibited a high antioxidant activity only in terms of the DPPH radical scavenging activity [87]. Parsley and lovage had a weak antioxidant activity, whereas basil showed a moderate antioxidant activity. Finally, thyme EO showed the highest antioxidant capacity [86].

Bergamot and lemon EOs extracted from the fruit peel of several citrus varieties were analyzed to determine their antioxidant activity through a thiobarbituric acid reactive substances (TBARS) test in a fish model (sardine). Samples of sardine treated with the bergamot EO displayed greater antioxidant activity than lemon EO [75].

### 4.3. Immunomodulatory Activity Effects of Essential Oils in Cells and Animals

Inflammation is a complex immune response against different types of harmful factors. Pathogenic microorganisms, irritants compounds, or damaged tissue induce an acute inflammatory response that can persist for a short period of time, which is beneficial for the host. In spite of this, if resolution of the inflammation is not adequate or the stimulus persists, then it is called chronic inflammation, which predisposes the hosts to different diseases such as cancer, cardiovascular disease, neurological disease, and metabolic disorders. During a chronic inflammation response, different signaling pathways are activated, leading to the overexpression of pro-inflammatory genes and proteins such as the NF-κB transcription factor and cytokines including IL and TNF-α. This inflammation is also related to an increased release and accumulation of ROS and reactive nitrogen species (RNS). When ROS production is greater than the cellular antioxidant capacity, oxidative stress can harm lipids, proteins, and DNA [111]. In this sense, EOs are of the greatest interest because of their anti-inflammatory and antioxidant properties, which are a potential source for the development of functional foods.

In general, EOs did not produce any cytotoxic effect when they were used at low concentrations; indeed, in human blood-isolated lymphocytes from healthy donors, *Pistacia vera* L. EOs significantly increased cell viability [64]. However, a high dose can have a negative effect on cell viability. In the case of malignant cells, it has been described that EOs derived from *Heracleum pyrenaicum* subsp. *orsinii* inhibited cell growth, which is in agreement with the established criteria from the National Cancer Institute (NCI), whereas they showed no toxic side effects on normal MRC-5 cells [69]. In line with these results, *Pituranthos tortuosus* EO is able to inhibit cell proliferation in a concentration-dependent and time-dependent manner on B16F10 melanoma cancer cells, which is likely by an increased apoptotic pathway [91]. Likewise, in human colonic adenocarcinoma cancer cell lines (HT29-D4 and Caco-2 cell), *Allium roseum* L. EO has a growth-inhibitory effect in a dose-dependent manner, without being cytotoxic. This effect has been attributed to the presence of sulfurous compounds as the major constituents of this EO [68]. Conversely, *Cirsium japonicum* DC EOs could promote cell proliferation in the human pulmonary adenocarcinoma A549 cell line [92].

On the other hand, in LPS-stimulated murine macrophage RAW264.7 cells, treatment with EOs derived from *Trachydium roylei*, *Artemisia argyi*, and *Chmaecyparis obtusa* has been shown to inhibit the secretion of pro-inflammatory cytokines, whereas treatment with EOs derived from *Trachydium roylei* also increased the secretion of IL-10, which is an anti-inflammatory cytokine. Therefore, the regulation of cytokines in this cell model may be one of the mechanisms by which EOs have an anti-inflammatory effect [22,90,94,95]. In the case of *Artemisia argyri* EOs, the regulation of NF-κB and AP-1 translocation has been proposed as a possible mechanism for its anti-inflammatory effect. In addition, a significant phosphorylation of JAK2 and STAT1/3 was also observed, but not the activation of NF-κB and mitogen-activated protein kinase (MAPK) cascades [22]. Other important mediators in inflammation are the production of nitric oxide (NO), secretion of prostaglandin E2 (PGE2), and the production of ROS. EOs have been shown to affect the expression of inducible nitric oxide synthase (iNOS) and cycloxygenase-2 (COX-2) expression; therefore, they might affect the secretion of NO and PGE2. In line with this, *Artemisia argyri* and *Trachydium roylei* EOs have been described to alter iNOS and COX-2 gene and protein expression, and to inhibit NO and PGE2 secretion and ROS production [22,95]. In immune human cells, there are studies showing that EOs exert their anti-inflammatory effects through the regulation of cytokine secretion and ROS production [31,93].

Similarly, in C57BL/6 mice, treatment with EOs has been shown to be efficient in reducing the levels of pro-inflammatory mediators [89,94]. In the contact hypersensitivity response, treatment with *Litsea cubea* L. EOs was able to inhibit the immune response [89]. In one interesting article, Sutili et al. described the use of *Hesperozygis ringes* and *Ocimun americamun* in silver catfish exposed to *Aeromonas hydrophila*, where this EO significantly decreases the hematocrit values and increases the plasma cortisol level and complement system activity. These results indicated a potential use of EOs in the treatment of infected fish [101]. 

One possible use of EOs is in poultry production as a supplement in the diet to improve production and to decrease the use of antibiotics. Their use in broiler chicken has been shown to improve animal growth. One interesting point is the effect of EOs in gastrointestinal microbiota composition, where supplementation with them has been shown to exert a positive effect—decreasing the pathogenic microorganism while increasing the number of probiotic bacteria such as *Lactobacillus* spp. [96,97,98]. Correspondingly, in weaned piglets, EOs or their main active compounds positively modulated gastrointestinal microbiota [23,99,100]. In addition, the use of carvacrol and thymol enriched protein biosynthesis, amino acids, and lipid metabolism [100].

Owing to this, EOs could be useful to inhibit pathogenic bacteria without affecting gastrointestinal commensal bacteria. Using static batch culture systems inoculated with human feces, Thapa el al. have shown that several EO compounds selected for their effectiveness against gastrointestinal pathogen need not have a toxic outcome on commensals bacteria at concentrations that would probably suppress pathogen bacterial growth. In this regard, the relative proportion of bifidobacteria was increased, while *Bacteroidetes* and *Clostridium* clusters IV and XIVa were not significantly affected. In terms of fermentation, except for high concentrations of thymol and geraniol, the essential oil compounds had no effects [112].

## 5. Conclusions

EOs have important antimicrobial and immunomodulatory properties that make them suitable for food preservation, alternatives to antibiotics, and ingredients in functional foods. In this regard, antimicrobial activity was tested in several strains with a wide range of observed results; the inhibition of *S. aureus* and even *V. cholerae* and *C. albicans* has been reported. Some EOs have demonstrated their efficacy against several foodborne pathogens in vitro and model food systems, and they can be applied in foods to improve their microbiological safety; however, these aforementioned results cannot be always extrapolated. One important effect of EOs is their antioxidant properties, with a dose range between 0.01 and 10 mg/mL. The EOs have demonstrated remarkable antioxidant activities, which can also be attributed to their richness in terms of phenolic derivatives.

Concerning their immunomodulatory effect, most of the articles highlighted that EOs did not produce a cytotoxic effect when they are used at low concentrations. Their immunomodulatory activity can be attributed to their ability to modify the secretion of cytokines, which is probably through the regulation of NF-κB, but also through the MAPK signaling pathway, or through their ability to affect the cellular expression of iNOS and the secretion of prostaglandins. 

Although the biological properties of EOS have been studied, there is a need for more well-designed studies, involving a normalization of dose and incubation time in cell and animal models that will allow gaining a better understanding of their biological activities and underlying mechanisms. Well-designed studies in animals and humans are compulsory to evaluate the efficacy of EOs.

## Figures and Tables

**Table 1:** PICO (Population, Intervention, Comparison, Outcome) criteria for inclusion of studies.

Parameter	Inclusion Criteria
Population	Studies performed in cells and animals, including humans
Intervention	Treatment with essential oil
Comparison	Essential oil vs. control
Outcome	Antimicrobial, antioxidant, and immunomodulatory effects

**Table 2:** Main characteristics of studies related with antibacterial properties of essential oils.

Article	Plant Derived EOs	Main Components of EOs	Bacteria	MIC/MBC/IC_50_
Aghraz et al. [25]	*Cladanthus arabicus* and *Bubonium imbricatum*	*Cladanthus arabicus*: oxygenated monoterpenes (61.4%): cis-chrysanthenyl acetate (31.4%) and thymolisobutyrate (3.4%); *Bubonium imbricatum*: monoterpenes hydrocarbons (75.8%): sabinene (31.1%), β-pinene (16.7%), myrcene (12.3%), and α-pinene (5.3%)	*E. coli*, *K. pneumoniae*, *E. cloacae*, *P. mirabilis*, *Salmonella* spp.	MIC 200–800 μg/mL for *C. arabicus*, MIC 400–1600 μg/mL for *B. imbricatum*
Alarcon et al. [26]	*Ruilopezia bracteosa*	β-myrcene (34.2%), α-pinene (24.3%), 7-epi-α-selinene (9.1%), and β-pinene (8.5%)	*S. aureus*, *E. faecalis*, *K. pneumoniae*, *E. coli*, *S.* Typhi, *P. aeruginosa*	MIC 10 μg/mL
Ashraf et al. [27]	*Nigella sativa*	Hymoquinone, dithymoquinone, thymohydroquinone, and thymol	*S.* Enterica	MIC ≥1000.0 ± 322.7 μg/mL
Behbahani et al. [28]	*Oliveria decumbens*	Thymol (28.45%) γ-terpinene (22.2%), ρ-cymene (17.90%), myristicin (13.55%), carvacrol (8.50%), and limonene (2.60%)	*P. aerogenes*, *E. coli*, *S. pyogenes*, *S. epidermidis*	MIC, 1–8 mg/mL; MBC 1–16 mg/mL
Boonyanugomol et al. [29]	*Zingiber cassumunar*	sabinene, γ-terpinene, α-terpinene, terpinene-4-ol, and (E)-1–(3,4-dimethoxyphenyl)butadiene	*Acinetobacter baumannii*	MIC/MBC: 7.00–9.24 mg/mL
Chaib et al. [30]	*Asteriscus graveolens* and *Pulicaria incisa*	*Asteriscus graveolens:* cis-chrysanthenyl acetate (31.1%), myrtenyl acetate (15.1%), and kessane (11.5%); *Pulicaria incisa:* chrysanthenone (45.3%) and 2,6-dimethylphenol (12.6%)	*K. pneumoniae*, *E. coli*, *A. baumannii*, *P. aeruginosa*, *L. monocytogenes*, *S. aureus*, *P. mirabilis*	MIC: 19–1250 μg/mL
Chen et al. [31]	*Kunzea ericoides* and *Leptospermum scoparium*	-	*T. mucoides*, *C. tropicalis*, *S. aureus*, *S. mutans*, *S. sobrinus*, *E. coli*	MIC 0.78%–3.13%
Chiboub et al. [32]	*Foeniculum vulgare MILL*, *Daucus carota* L. subsp. *sativus*	*Daucus carota:* isospathulenol, caryophyllene oxide, and δ-elemene *Foeniculum vulgare MILL:* (E)-anethole p-anisaldehyde, p-acetonylanisole, limonen, *exo*-fenchol acetate, and methyl chavicol	*S. aureus*, *B. subtilis*, *B. amyloliquefaciens*, *S.* Enterica, *E. coli*, *C. albicans*	MIC: 6.25–50 mg/mL
Condo et al. [33]	*Pimpinella anisum* L., *Cinnamomum zeylanicum*, *Syzygium aromaticum*, and *Cuminum cyminum* L.	*Pimpinella anisum* L: trans-anethole ((E)-1-methoxy-4-(1-propenyl) benzene); *Cinnamomum zeylanicum:* cinnamaldehyde; *Syzygium aromaticum*: eugenol; *Cuminum cyminum:* cuminaldehyde (4-isopropylbenzaldehyde), and cuminyl alcohol (4-isopropyl-benzyl-alcohol)	*S. aureus*, *S. epidermidis*, *E. faecalis*, *S. pyogenes*, *E. coli*, *P. aeruginosa*, *A. hydrophila*, *P. mirabilis*, *K. pneumoniae*, *C. albicans*	
De Jesus et al. [34]	*Myrcia ovata Cambessedes*	Geranial (40%), neral (28%), citronella (9%)	*P. aeruginosa*, *S. aureus*, *B. cereus*, *B. subtilis*, *E. faecalis*, *S. marcescens*, *E. coli*, *S. enteritidis*	MIC: 0.78–25 μL/mL
Elshafie et al. [35]	*Verbena officinalis*, *Majorana hortensis*, and *Salvia officinalis*	*Verbena officinalis:* Isobornyl formate (45.4%), (E)-citral (47.5%); *Majorana hortensis*: 1,8-cineole (33.5%), β-phellandrene (9.1%), α-pinene (9%), limonene (6.4%); *Salvia officinalis:* Trans-thurjone (37.9%), canfor (13.9%), and borneol (7.6%)	*B. megaterium*, *B. mojavensis*, *C. michiganensis*, *E. coli*, *X. campestris*, *P. savastanoi*, *P. syringae pv. phaseolicola*	MIC: 1000–10,000 mg/L
Fadil et al. [36]	Mixture of *Thymus vulgaris*, *Rosmarinus officinalis* L., and *Myrtus communis* L.	*T. vulgaris:* Thymol (37.54%), p-cymene (14.49%), c-terpinene (11.15%), linalool (4.71%), and carvacrol (4.62%); *R. officinalis*: α-pinene (48.58%), 1,8-cineole (33.4%) and camphene (8.69%); *M. communis*: borneol (27.15%), 1,8-cineole (21.33%), α-pinene (11.09%), myrtenyl acetate (6.45%), trans-pinocarveol (4.82%), and α -terpineol (4.83%)	*S.* Typhimurium	Thyme MIC: 0.25% (*V*/*V*); myrtle MIC: 0.5% (*V*/*V*); Rosemary MIC: 2% (*V*/*V*)
Falsafi et al. [37]	*Satureja bachtiarica Bunge*	Carvacrol (45.5%), thymol (27.9%), p-cymene (4.4%), γ-terpinene (4.0%), α-pinene (1.5%), 1,8-cineole (1.3%), α-terpinene (1.2%), and E-caryophyllene (1.1%)	*H. pylori*	MIC: 0.035 μL/mL
Fournomiti et al. [38]	*Origanum vulgare*, *Salvia officinalis*, *Thymus vulgaris*	*Origanum vulgare:* Carvacrol and thymol; *Salvia officinalis*: 1,8-cineole, α-thujone and camphor; *Thymus vulgaris:* thymol and carvacrol	*E. coli*, *K. oxytoca*, *K. pneumoniae*	MIC oregano: 0.9 mg/mL; 73.5 µg/mL; MIC thyme: 8.1 µg/mL; 9.5 µg/mL; 28.6 µg/mL against *K. oxytoca*, *K. pneumoniae* and *E. coli*, respectively
Gadisa et al. [39]	*Blepharis cuspidata*, *Boswellia ogadensis*, and *Thymus schimper*		*E. coli*, *K. pneumoniae* and MRSA	MIC: 0.39–6.25 μL/mL/MBC (0.78–12.5 μL/mL) against *MDR E. coli* and *K. pneumoniae*
Igwaran et al. [40]	*Tagetes minuta*	β-Ocimene (14.40%), m-tert-butyl-Phenol (9.41%), 2,6-dimethyl-, (E)-5,7-Octadien-4-one (7.14%), 1,2, 3,4,4a,5,6,7-octa hydro-4a-methyl-naphthalene (5.58%), and spathulenol (4.56%)	*S. uberis*, *E. cloacae*, *S. aureus*, *M. smegmatis*, *L. ivanovii*, *Vibrio* spp., *E. coli*	MIC (*S. aureus*, *M. smegatis*, and *S. uberis*): 0.125 mg/mL; *L. ivanovii*, *Vibrio* spp., *E. cloacae* and *E. coli*: 0.06 mg/mL. MBC (*E. cloacae* and *E. coli*): 0.06 mg/mL; MBC *S. uberis:* 0.5 mg/mL; *Vibrio* spp.: 0.125 mg/mL
Jaradat et al. [41]	*Thymus bovei*	trans-geraniol (35.38%), α-citral (20.37%), β-citral (14.76%), cis-geraniol (7.38%), and 3-octanol (4.38%)	*S. aureus*, *E. coli*, *P. aeruginosa*, *C. albicans*	MIC: 0.25–0.5 mg/mL
Lee et al. [42]	Hibicuslide C	-	*P. aeruginosa* strains	MIC range: 5.0–10.0 µg/mL
Linde et al. [43]	*Petroselinum crispum*	Apiol (50.3%), myristicin (14.0%), and β-phellandrene (14.6%)	*B. cereus*, *E. cloacae*, *L. monocytogenes*, *E. coli*, *P. aeruginosa*, *S.* Typhimurium, *S. aureus*	MICs 0.04–1.0 mg/mL. MBCs 0.15–10.0 mg/mL
Mahmoudzadeh et al. [44]	*Carum copticum*	thymol (36.4%), p-Cymene (31.4%), and γ-Terpinene (21.73%)	*E. coli*	MIC 0.05%–1.75%; MBC 0.052%–3.25%
Man et al. [45]	*Boswellia sacra*, *Myrtus communis*, *Thymus vulgaris*, *Citrus limon*, *Origanum vulgare*, and *Lavandula angustifolia*	*Boswellia sacra:* β-pinene (25.6%), α-terpinene (18.6%); *Myrtus communis:* β-pinene (25%), eucalyptol (28.7%); *Thymus vulgaris:* linalool (56.5%), geranyl propionate (16.3%); *Citrus limon*: limonene (36.9%), β-pinene (15%), α-pinene (19.2%); *Origanum vulgare:* carvacrol (80.5%); *Lavandula angustifolia:* linalyl-butyrate (26.5%), and linalool (25%)	*S. aureus*, *E. faecalis*, *E. coli*, *K. pneumoniae*, *P. aeruginosa*	MICs/MBCs 0.1% to >50%
Marrelli et al. [46]	*Origanum dictamnus*, *Origanum libanoticum* and *Origanum microphyllum*	*O. dictamnus*: p-cymene (32.7%), γ-terpinene (12.4%), carvacrol (14.7%), and linalool (7.8%); *O. microphyllum*: Terpinen-4-ol (16.2%), carvacrol (13.3%), sabinene (7.5%), and trans-sabinene hydrate (7.1%); *Origanum libanoticum*: linalool (6.5%), thymol methyl ether (9.8%), (E)-b-caryophyllene (7.7%), and hexadecanoic acid (11.3%)	*B. cereus*, *B. subtilis*, *S. epidermidis*, *S. aureus*, *S. faecalis*, *E. coli*	*O. dictamnus* MIC: 25–50 mg/mL
Meng et al. [47]	*Juniperus rigida*	Caryophyllene (13.11%) and α-caryophyllene (11.72%)	*K. pneumoniae*	MIC/MBC: 3.125 mg/mL
Montironi et al. [48]	*Minthostachys verticillata*	Pulegone (51.7%) and menthone (37.8%)	*S. uberis*	MIC: 14.3–114.5 mg/mL/MBC: 114.5–229 mg/mL
Mutlu-Ingok et al. [49]	Cardamom, cumin, and dill weed	Cumin: p-mentha-1,3-dien-7-al (26.7%), cumin aldehyde (24.1%), γ-terpinene (16.9%), and β-pinene (14.4%); Cardamom: *α*-terpinly acetate (43.4%) and 1,8-cineole (29.2%); Dill weed: carvone (41.6%), and limonene (27.4%)	*C. jejuni*, *C. coli*	MIC/MBC: 0.05 L/mL, cumin, Cardamon/cumin MIC/MBC: 0.025 L/mL
Okoh et al. [50]	*Peperomia pellucida*	Linalool, d-limonene, β-caryophyllene, and linalyl acetate were the major compounds	*S. aureus*, *L. ivanovii*, *M. smegmatis*, *S. uberis*, *E. cloacae*, *E. coli*, *V. paraheamolyticus*	MIC: 0.15–0.20 mg/mL
Okoh et al. [51]	*Jatropha gossypifolia*	Phytol, germacrene D, 𝛼-copaene, 𝛼-terpinene, and limonene were the major compounds	*E. coli*, *E. faecium*, and *S. aureus*	MIC/MBC: 0.025–0.10 mg/; MBC: 0.05–0.10 mg/mL
Oukerrou et al. [52]	*Aloysia citriodora*	𝛽-spathulenol (15.61%), ar-curcumene (14.15%), trans-caryophyllene oxide (14.14%), and neral (10.02%)	*E. coli*, *S. aureus*, *P. aeruginosa*	MIC: 2.84–8.37 mg/mL
Paredes et al. [53]	*Senecio nutans*	Methyl cinnamate (44.9%), p-cymenol (27.2%),	*Vibrio cholerae*	MIC: 0.4 mg/mL
Patra et al. [54]	*Enteromorpha linza*	Hexadecanoic acid, nonadecadiene, tetradecanoic acid, tridecanol, and azetidine	*B. cereus*, *S. aureus*	MIC/MBC: 12.5–25 mg/mL
Pereira et al. [55]	*Baccharis dracunculifolia*	-	*Streptococcus mutans*	MIC: 6%
Porfirio et al. [56]	*Lippia alba*	Geranial, neral, p-cymene, geranic acid, carvone, and limonene were the major compounds	*S. aureus*	MIC 0.5–1 mg/mL; MBC: 0.5–2 mg/mL
Puškárová et al. [57]	*O. vulgare*, *T. vulgaris*, *S. sclarea*, *L. angustifolia*, *E. Caryophyllata*, and *T. plicata*	-	*E. coli*, *S.* Typhimurium, *Y. enterocolitica*, *S. aureus*, *L. monocytogenes*, *E. faecalis*, *B. cereus*, *A. protophormiae*, *P. fragi*	MIC/MBC: 0.025%–0.5%
Sakkas et al. [58]	*Ocimum basilicum* L., *Matricaria chamomilla*, L.*Thymus capitatus*, L., Me*laleuca alternifolia*, *Thymus vulgaris*, L.	*Ocimum basilicum L.:* estragole; *Matricaria chamomilla*, *L.:* bisabolol and trans-b-farnesene; *Thymus capitatus*, *L.:* carvacrol and thymol; *Melaleuca alternifolia*: terpinen-4-ol and p-cymene; *Thymus vulgaris*, *L:* thymol, p-cymene, and linalool	*A. baumannii*, *E. coli*, *K. pneumoniae* and *P. aeruginosa*	MIC/MBC values ranged from 0.12% to 1.50% (*v*/*v*) for tea tree oil, 0.25%–4% (*v*/*v*) for origanum and thyme oil, 0.50% to >4% for basil oil
Salem et al. [59]	*Cupressus macrocarpa Hartw* and *Corymbia citriodora (Hook.)*	Terpinen-4-ol (23.7%), α-phellandrene (19.2%), α-citronellol (17.3%), and citronellal were the major constituents of *C. macrocarpa*, and α-citronellal (56%), α-citronellol (14.7%), citronellol acetate (12.3%), isopulegol, and eucalyptol were the primary constituents of *C. citriodora*	*B. cereus*, *L. monocytogenes*, *M. flavus*, *S. aureus*, *D. solani*, *E. coli*, *P. atrosepticum*, *P. carotovorum* subsp*. Carotovorum*, *P. aeruginosa*	MIC *C. citriodora* leaves 0.06–0.20 mg/mL, MBC: 0.12–0.41 mg/mL; MIC *C. macrocarpa*: 0.07–0.31 mg/mL, MBC: 0.15–0.63 mg/mL
Semeniuc et al. [60]	Parsley, lovage, basil, and thyme	β-myrcene, β-phellandrene, γ-terpinene, and α-pinene were the major compounds	*B. cereus*, *S. aureus*, *P. aeruginosa*, *E. coli*, *S.* Typhimurium	*B. cereus* MIC Basil: 10.8 μL/mL; thyme: 0.56 μL/mL; *S. aureus* MIC Basil: 2.45 μL/mL and thyme 0.06 μL/mL. *P. aeruginosa* MIC Basil 10.80 μL/mL and thyme 0.27 μL/mL. *S.* Typhimurium MIC Basil: 22.68 μL/mL and thyme: 0.56 μL/mL
Sharafiti Chaleshtori et al. [61]	*Bunium persicum*, *Eucalyptus globulus*, and rose water	*B. persicum*, β-pinene (11.72%), p-cymene (15.47%), g-terpinene (18.32%), cumin aldehyde (38.4%), p-mentha-1,3-dien-7-al (5.37%), and p-mentha1,4-dien-7-al (2.86%); *E. globulus*, limonene (9.4%) and 1,8-cineole (70.3%); rose water, linalool (6.6%), terpineol (3.3%), carvone (0.31%), citronellol (6.85%), trans-geraniol (7.11%), phenylethanol (66.84%), eugenol (4.53%), cytronellol, hydroxyl (1.15%), and geranic acid (1.2%)	*Listeria* spp.	MIC: 0.351–2.812 mg/mL MBC: 0.703–5.625 mg/mL
Sharifi et al. [62]	*Thymus daenensis*; *Satureja hortensis*	*T. daenensis:* carvacol (40%–69%), followed by γ-terpinene (30%–28%) and α-terpinene (5%–52%)*; S. hortensis:* thymol (41%–28%), γ-terpinene (37%–63%), p-cymene (2%–12%) and α-terpinene (3%–52%)	*S. aureus*	MICs of *T. daenensis*: 0.0625 μL/mL; *S. hortensis* 0.125 μL/mL; MBC 0.125 μL/mL
Sharifi-Rad et al. [63]	*Xanthium strumarium* L.	cis-β-guaiene (34.2%), limonene (20.3%), borneol (11.6%), and bornyl acetate (4.5%)	*K. pneumoniae*, *E. coli*, *P. aeruginosa*, *S. aureus*, *S. epidermis*, *B. subtilis*	MIC *S. aureus:* 0.5 μg/mL; MIC *B. subtilis* 1.3 μg/mL; MIC *K. pneumoniae* 4.8 μg/mL
Smeriglio et al. [64]	*Pistacia vera* L.	4-Carene, α-pinene, and δ-3-Carene were the major compounds	*S. aureus*, *S. aureus* MRSA, three clinical isolates of *S. aureus*, *E. coli* and *P. aeruginosa*	MIC/MBC: 7.11 mg/mL inhibited the growth of all the tested strains, with the exception of *Pseudomonas*
Snoussi et al. [65]	*Petroselinum crispum*, *Ocimum basilicum*	*P. crispum:* 1,3,8-p-menthatriene (24.2%), β-phellandrene (22.8%), apiol (13.2%), myristicin (12.6%), and terpinolene (10.3%); *O. basilicum*: linalool (42.1%), (E)-methylcinnamate (16.9%), and 1,8-cineole (7.6%)	*V. alginolyticus*, *V. alginolyticus*, *V. parahaemolyticus*, *V. parahaemolyticus*, *Vibrio vulnificus*, *V. vulnificus*, *V. cholerae*, *A. hydrophila*	*P. crispum*: MIC: 0.011–0.044 mg/mL MBC:2.81–11.25 mg/mL; *O. basilicum* MIC 0.019–0.039 mg/mL; MBC 2.5–10 mg/mL
Soliman et al. [66]	*Calligonum comosum*	Benzaldehyde derivative was the major compound	*P. aeruginosa*, *K. pneumoniae*, *A. baumannii*, and *E. coli*	MIC: 180.0–200.0 µg/mL
Tibyangye et al. [67]	*Ocimum suave*	Linalool and geraniol were the major compounds	*E. coli*, *K. pneumoniae*, *S. aureus*, *E. faecalis*, *M. morganii*, *Citrobacter* spp., *Enterobacter* spp. and *P. aeruginosa*	MIC: 0.78–22 μg/mL
Touihri et al. [68]	*Allium roseum*	Methyl methanethiosulfinate, 3-vinyl-1,2-dithiacyclohex-5-ene, and diallyl trisulfide were the major compounds	*S. aureus*, *K. pneumoniae*, *E. coli*, *E. faecalis*, *S.* Typhimurium	MIC: 0.078–2.5 mg/mL
Ušjak et al. [69]	*Heracleum pyrenaicum* subsp*. orsinii (Guss.)*	β-pinene, (Z)-β-ocimene, and α-pinene were the major compounds	*S. aureus*, *B. cereus*, *L. monocytogenes*, *M. flavus*, *P. aeruginosa*, *E. coli*, *S.* Typhimurium, *E. cloacae*	*B. cereus* (MIC: 0.21 mg/mL, MBC: 0.53 mg/mL*). S.* Typhimurium, *E. coli*, *P. aeruginosa* (MICs: 0.23 mg/mL, MBCs: 0.47 mg/mL), *S. aureus* (MIC: 0.23 mg/mL, MBC: 0.47 mg/mL)
Utegenova et al. [70]	*Ferula ovina (Boiss.)*	α-pinene (47.8%), β-pinene (7.1%), sabinene (20.5%), β-phellandrene (6.5%), and trans-verbenol (7.4%)	MRSA	IC_50_: 19.1–22.9 μg/mL
Vieira et al. [71]	*Eucalyptus globulus*, *Thymus mastichina* L., *Mentha pulegium* L., *Rosmarinus officinalis* L., *Calamintha nepeta*, *Cistus ladanifer* L., *Foeniculum vulgare* L., *Dittrichia viscosa*	*Lamiaceae:* isopulegol, isopulegone and 1,8- Cineole; *C. nepeta:* pulegone; *M. pulegium*: β-myrcene, camphor, 1,8-cineole; *R. officinalis:* α-pinene, and 1,8-cineole; *T. mastichina:* α-terpinyl acetate*; C. ladanifer*: α-pinene, camphene, fenchone, bornyl acetate, and viridiflorol*; E. globulus:* 1,8-cineole; *F. vulgare*: anethol, b-myrcene and fenchone; *D. viscosa:* E-nerolidol and fokienol	*S. aureus*, *B. subtilis*, *E. coli*, *P. aeruginosa*	MIC: 6–25 mg/mL
Xu et al. [72]	*Syringa yunnanensis*	Eugenol (76.23%), β-caryophyllene (11.54%), caryophyllene oxide (4.29%), and eugenyl acetate (1.76%)	*S. aureus*	MIC: 0.625 mg/mL
Zhao et al. [73]	*Fagopyrum esculentum*, *Fagopyrum tataricum*, *Fagopyrum Cymosum*	*F. esculentum:* nonanoic acid (7.58%), (E)-3-hexen-1-ol (6.52%), benzothiazole (5.08%), 2-Pentadecanone (18.61%), eugenol (17.18%); *F. tataricum:* 1,2-benzenedicarboxylic acid, bis(2-methylpropyl) ester (13.19%), and (E,E)-farnesylacetone (7.15%); *F.Cymosum:* eugenol (12.22%), (E)-3-hexen-1-yl acetate (8.03%), linalool oxide (7.47%), 1-hexanol (7.07%), and benzothiazole (6.72%)	*A. tumefaciens*, *E. coli*, *P. lachrymans*, *X. vesicatoria*, *B. subtilis*, *S. aureus*	MIC: 100.0–800.0 g/mL

Abbreviations: EO, essential oil; MIC, minimal inhibitory concentration; MBC, minimal bactericidal concentration; IC_50_ half-maximal inhibitory concentration; MRSA, methicillin-resistant *S. aureus.*

**Table 3:** Main characteristics of studies related with antioxidant properties of essential oils.

Article	Plant Derived EOs	Main Components of EOs	Method	Antioxidant Effects
Bag et al. [74]	Bay leaf, black pepper, coriander, cumin, garlic, ginger, mustard, onion, and turmeric	Coriander and cumin seed oil, linalool, p-coumaric acid	DPPH method	Coriander 150.62 (µg/mL), cumin 163.50 (µg/mL), mustard 155.16 (µg/mL)
Djenane et al. [75]	Orange (*Citrus sinensis* L.), lemon (*Citrus limonum* L.), and bergamot (*Citrus aurantium* L.) from Algeria	Limonene (77.37%) for orange EO; linalyl acetate (37.28%), linalool (23.36%) for bergamot EO; and limonene (51.39%), β-pinene (17.04%), and γ-terpinene (13.46%) for lemon EO	Antioxidant effect in treated sardine	A reduction of 2.50 log10 CFU/g was recorded during the third day of storage
Ehsani et al. [76]	*Melissa officinalis* and *Deracocephalum moldavica*	*M. officinalis*, citronellal, thymol, and citral; *D. moldavica*, geraniol, geranial, geranyl acetate, and neral	DPPH, BCBT, and ABTS assay	Both EOs showed strong activity in the maintenance of β-carotene molecules, which was higher than that of ascorbic acid
Hu et al. [77]	*Cyperus rotundus* L.	α–pinene, cyperene, α–cyperone, and cyperotundone were the major compounds	DPPH and ABTS radicals	DPPH radicals were far lower than that of Trolox (13.1 μg/mL); however, ABTS radicals were significantly higher than Trolox (84.7 μg/mL)
Jaradat et al. [78]	*Ruta chalepensis*	Linalyl acetate, 𝛽-linalool, 2-undecanone, and 2-nonanone were the major compounds	DPPH method	Percentages of inhibition for *R. chalepensis* collected from Jerusalem, Hebron, and Jenin were 6.9 ± 0.94 µg/mL, 69.56%; 7.8 ± 1.05 µg/mL, 61.53%; and 19.9 ± 0.68 µg/mL, 24.12%, respectively
Kazemi et al. [79]	*Achillea millefolium* L., *Anethum graveolens* L., and *Carum copticum* L.	*A. millefolium*, thymol, carvacrol, borneol, and limonene; *A. graveolens*, thymol, limonene, α-pinene; and *C. copticum*, thymol, sabinene, and borneol	DPPH, FRAP, and BCBT assays	*A. millefolium* had the highest antioxidant activity in all conducted assays
Marin et al. [80]	*Foeniculum vulgare*, *Petroselium crispum*, and *Lavandula officinalis*	*L. officinalis*, linalool, and linalyl acetate; *F. Vulgare*, limonene, anethole, and fenchone; *P. crispum*, myristicin, α-pinene, and β-pinene	DPPH and FRAP assays	*P. crispum* presented the best antioxidant profile, given its highest % of inhibition of DPPH radical (64.28%) and FRAP (0.93 mmol/L Trolox)
Marrelli et al. [81]	Six different populations of *Origanum heracleoticum* L.	Limonene, carvacrol-methyl-ether, and carvacrol were the major compounds	DPPH and BCBT assays	Samples showed a modest DPPH value of 320.9 μg/mL, and BCBT of 4.68 μg/mL.
Okoh et al. [82]	*Dennettia tripetala* G. Baker	2-Methylphenyl formate, α–terpinene, and caryophyllene were the major compounds	DPPH, ABTS, nitric oxide, and lipid peroxyl	The EOs demonstrated strong ability in ABTS, lipid peroxide, and nitric oxide radical assays in a concentration-dependent manner
Okoh et al. [51]	*Jatropha gossypifolia* L.	Phytol, germacrene D, 𝛼-copaene, 𝛼-terpinene, and limonene were the major compounds	DPPH, ABTS, nitric oxide, and lipid peroxyl	The stem showed that the antiradical strength was superior to leaf EO
Okoh et al. [50]	*Peperomia pellucida* (L.) Kunth	Linalool, d-limonene, β-caryophyllene, and linalyl acetate were the major compounds	DPPH, ABTS, nitric oxide, and lipid peroxyl	The EOs demonstrated strong ability in DPPH, ABTS, nitric oxide and lipid peroxyl assays in a concentration-dependent manner
Ouedrhiri et al. [83]	*Ormenis mixta* and *Pelargonium asperum*	*P. asperum*, citronellol, citronellyl formate, and geraniol; *O. mixta*, germacrene, 1,8 cineol, and cis-methyl isoeugenol	DPPH method	*O. mixta* exhibited an important antioxidant activity, which was significantly higher than that exhibited by *P. asperum*
Pirbalouti et al. [84]	*Ferulago angulata*	α-pinene, and cis-β-ocimene were the major compounds	DPPH method	The highest antioxidant activity was obtained from the oil of the Kallar population (488 µg/mL) and butylhydroxyanisole as a positive control (321 µg/mL)
Poaty et al. [85]	Balsam fir, black spruce, white spruce, tamarack, jack pine, eastern white cedar, and Labrador tea EOs	α–pinene, β-pinene, δ-3-carene, and limonene were the major compounds. α–thujone was the main compound in white cedar	DPPH, FRAP assays	DPPH (concentration providing 50% inhibition ≥7 mg/mL)
Semeniuc et al. [86]	Parsley, lovage, basil, and thyme EOs	β-myrcene, β-phellandrene, γ-terpinene, and α-pinene were the major compounds	TEAC assay	The highest antioxidant capacity was found in thyme oil
Shakeri et al. [87]	*Glycyrrhiza triphylla* Fisch. and C.A.Mey	β-caryophyllene, limonene, and myrcene were the major compounds	The DPPH, and β-Carotene/linoleic acid assay	The oil was considerably active in the DPPH assay (100.40 ± 0.03 µg/mL)
Sharafati Chaleshtori et al. [61]	*Bunium persicum*, *Eucalyptus globulus*, and rose water	*B. persicum*, β-pinene (11.72%), p-cymene (15.47%), gterpinene (18.32%), cumin aldehyde (38.4%), p-mentha-1,3-dien-7-al (5.37%), and p-mentha1,4-dien-7-al (2.86%); E*. globulus*, limonene (9.4%) and 1,8-cineole (70.3%); rose water, linalool (6.6%), terpineol (3.3%), carvone (0.31%), citronellol (6.85%), trans-geraniol (7.11%), phenylethanol (66.84%), eugenol (4.53%), cytronellol, hydroxyl (1.15%), and geranic acid (1.2%)	FRAP	*Bunium persicum* EO showed the greatest antioxidant activity
Smeriglio et al. [64]	*Pistacia vera* L.	4-carene, α-pinene, and δ-3-carene were the major compounds	Determination of total phenolic compounds, DPPH, TEAC, FRAP, chelating capacity on Fe^2+^, BCBT assays, superoxide anion (O^2−^) scavenging assay and hydroxyl radical (−OH) scavenging assay	The *Pistacia vera* L. variety Bronte showed a strong iron-chelating activity and was found to be markedly active against hydroxyl radical, while little effect was found against the DPPH method
Snoussi et al. [88]	*Mentha spicata*	Limonene, 1,8-cineole, and carvone were the major compounds	DPPH method, reducing power, chelating power, and BCBT assays	DPPH IC_50_ 3.08 ± 0.07, reducing power EC_50_, 2.49 ± 0.07, chelating power IC_50_, 6.33 ± 0.12, and BCBT 6.4 ± 0.07
Salem et al. [59]	*Cupressus macrocarpa* and *Corymbia citriodora*	Terpinen-4-ol (23.7%), α-phellandrene (19.2%), α-citronellol (17.3%), and citronellal were the major constituents of *C. macrocarpa*, and α-citronellal (56%), α-citronellol (14.7%), citronellol acetate (12.3%), isopulegol, and eucalyptol were the primary constituents of *C. citriodora*	Standard butylhydroxytoluene	*C. citriodora* was higher than that of the positive control but lower than that of the standard, butylhydroxytoluene
Zhao et al. [73]	*Fagopyrum esculentum*, *Fagopyrum tataricum*, and *Fagopyrum Cymosum*	*F. esculentum:* Nonanoic acid (7.58%), (E)-3-hexen-1-ol (6.52%), benzothiazole (5.08%), 2-Pentadecanone (18.61%), and eugenol (17.18%); *F. tataricum:* 1,2-benzenedicarboxylic acid, bis(2-methylpropyl) ester (13.19%) and (E,E)-farnesylacetone (7.15%); *F.Cymosum:* Eugenol (12.22%), (E)-3-hexen-1-yl acetate (8.03%), linalool oxide (7.47%), 1-hexanol (7.07%), and benzothiazole (6.72%)	DPPH and BCBT assays	Three EOs have a similar antioxidant capacity in both evaluated methods

Abbreviations: EOs: essential oils; ABTS, 2,2′-azino-bis(3-ethylbenzothiazoline-6-sulphonic acid); BCBT, β-carotene bleaching test; CFU, colony-forming unit; DPPH, (2,2-diphenyl-1-picrylhydrazyl); FRAP, ferric-reducing antioxidant power; TEAC, Trolox equivalent antioxidant capacity.

**Table 4:** Main characteristics of studies related with immunomodulatory properties of essential oils in cells.

Author	Cell Line	Plant Derived EOs	EOs Concentration	Main Components of EOs	Exposure Time	LPS Stimulation	Main Results
Chen et al. [31]	THP-1 human monocyte/macrophage cell line	Kanuka and manuka	0.1–0.5–1–5–10%		48 h	yes (20 µg/mL)	EOs have no major toxic side effects on THP-1 cells. Kanuka and manuka EOs reduced the LPS-induced TNF-α secretion but have no effect on IL-4 secretion. Kanuka and manuka EO have no effect on unstimulated THP-1 cells.
Chen et al. [89]	C57BL/6 mouse bone marrow-derived dendritic cells (DCs)	*Litsea cubea* L.	1–2–4 × 10^5^- and 5 × 10^4^-fold dilution	Terpene aldehydes (75.09%) were the most abundant compounds	Cytotoxicity assay: 24 h; TNF-α assay: 6 h; IL-12 assay: 12 h	yes (100 ng/mL)	A slight cytotoxic effect was observed at 5 × 10^4^ -fold diluted EO. Release of TNF-α and IL-12 by LPS-induced DCs were inhibited by EO in a dose-dependent fashion (IC_50_ of 1 × 10^5^- and 2 × 10^5^-fold dilution for TNF-α and IL-12, respectively).
Chen et al. [22]	Murine macrophage RAW264.7 cells	*Artemisia argyi*	270, 90, 30, and 10 µg/mL)	Cineole, camphor, (−)-borneol, and α-(−)-thujone were the major compounds	16 h	yes (1 µg/mL)	In LPS-induced cells, the EOs inhibited the release of NO, PGE_2_, and ROS and TNF-α, IL-6, IFN-β and MCP-1. In addition, EOs downregulate the gene and protein expression of iNOS and COX-2 without affecting its enzymatic activity. EOs also inhibited the phosphorylation of JAK2 and STAT1/3 but did not affect the MAPK and NF-κB cascades.
Cheng et al. [90]	Murine macrophage RAW264.7 cell	Oregano (*Origanum vulgare* L.)	≤10 μg/mL	Carvacrol and thymol were the major compounds	12 h	yes (1 µg/mL)	Low dose of EOs (1.25–20 μg/mL) did not produce any toxicity. In LPS-induced RAW264.7 cells, pretreatment with the EOs reduced the expression and secretion of IL-1β, IL-6, and TNF-α. Inhibition of LPS-induced MAPK, PKB, and NF-κB was also observed. The EOs also inhibited the LPS-induced elevation of NADPH oxidase and oxidative stress
Krifa et al. [91]	Splenocyte suspension from Balb/c mice; Murine melanoma B16F10 cell line	*Pituranthos tortuosus*	Splenocyte suspension: 1.25, 2.5, 5, and 10 μg/mL. B16F10 cell line: 25, 50, 100, 200, and 400 μg/mL.	Sabinene, α-pinene, limonene, and terpinen-4-ol were the major compounds	48 h	yes (5 μg/mL)	EOs treatment was able to promote LPS-stimulated splenocyte proliferation. In B16F10 cells, incubation with the EOs inhibited cell proliferation in a dose- and time-dependent fashion (IC50: 80 μg/mL). In addition, EOs treatment was also able to increase the number of apoptotic cells.
Ma et al. [92]	L02 cell line; Human lung adenocarcinoma A549 cell line; Murine macrophage RAW264.7 cell	*Cirsium japonicum* DC	25, 50, 100, and 200 μg/mL	Flavonoids, saponins, polysaccharides EO, coumarin, and alkaloids	24 h	yes (1 µg/mL)	EOs have no major toxic side effects on L02 cells, and even promoted cell proliferation. In the A549 cell line, EOs promote the proliferation of cancer cells. NO production was inhibited in LPS-induced RAW264.7 cells treated with EOs at 50 and 100 μg/mL. In addition, EOs treatment reduces the secretion of IL-6, but has no effect on TNF-α gene expression. Furthermore, EOs decreased lipid accumulation in ox-LDL-induced RAW264.7 cell, and decreased the secretion of IL-6.
Marelli et al. [81]	Murine macrophage RAW264.7 cells	*Origanum heracleticum* L.	25–1000 μg/mL	Limonene, carvacrol-methyl-ether, and carvacrol were the major compounds	24 h	yes (1 µg/mL)	In LPS-stimulated RAW264.7 cells, all EOs from *Origanun heracleticum* L. showed anti-inflammatory activity by means of its capacity to decrease the NO production.
Özek et al. [93]	Human blood isolated neutrophils from healthy donors; bone marrow leukocytes isolated from Balb/c mice	*Ferula iliensis*	1%	(E)-Propenyl sec butyl disulfide, (Z)-Propenyl sec butyl disulfide, and 10-Epi-g-eudesmol were the major compounds	Ca^2+^ flux assay: 0.06 h; ROS production: 0.5 h	no	EOs activated human neutrophil Ca^2+^ flux; this activation was dose-dependently inhibited by capsazepine, a TRPV1 channel antagonist. This effect was confirmed on TRPV1 channel-transfected HEK293 cells. Furthermore, EOs also activated SOD-inhibitable ROS production in both human neutrophils and mouse bone marrow phagocytes.
Park et al. [94]	Murine macrophage RAW264.7 cells	*Chamaecyparis obtusa*	1, 10, 50, and 100 μg/mL	α-terpinyl acetate, β-phellandrene, β-myrcene, limonene, bornyl acetate, γ-terpinene, β-thujaplicin, and α-terpineol	1 h	yes (1 µg/mL)	In LPS-stimulated cells, EOs treatment reduced nitric oxide, TNF-α, and IL-6 production, and inhibited iNOS and COX-2 expression.
Puskárova et al. [57]	human embryo lung HEL12469 cells	*Origanum vulgare*; *Thymus vulgaris*; *Salvia sclarea*; *Lavandula angustifolia*; *Eugenia caryophyllata*; and *Thuja plicata*	0.0025–1.0 μL/mL	-	24 h	no	EOs present toxic side effects at higher concentrations. Treatment with EOs did not induce any significant increase in DNA strand breaks; only *Thuja plicata* EO (0.2 μL/mL) showed a negative effect on DNA single-strand breaks in HEL 1269 cells.
Smeriglio et al. [64]	Human blood isolated lymphocytes from healthy donors	*Pistacia vera* L.	20, 17.5, 15, 12.5, 10, 7.5, 5, and 1 μg/mL	4-Carene, α-pinene, and δ-3-carene were the major compounds	24 h	no	EOs did not show any cytotoxic effects. In tert-butyl hydroperoxide-treated lymphocytes, incubation with EOs (20–12.5 μg/mL) significantly increased cell viability.
Touihri et al. [68]	Human colonic adenocarcinoma HT29-D4 and Caco-2 cell lines	*Allium roseum* L.	10, 20, 30, and 40 μg/mL	Methyl methanethiosulfinate, 3-vinyl-1,2-dithiacyclohex-5-ene, and diallyl trisulfide were the major compounds	Cytotoxicity assay: 5 h; Proliferation assay: 72 h	no	EOs did not show cytotoxic effects. Antiproliferative assay depicted that the number of cells was reduced by the incubation of HT29-D4 and Caco-2 cells with EOs in a dose-dependent fashion.
Ušjak et al. [69]	Human cervix Hela cell; human colon carcinoma LS174 cell; non-small cell lung carcinoma A549; human normal fetal lung fibroblast MRC-5 cell	*Heracleum pyrenaicum* subsp. o*rsinii*	12.5, 25, 50, 100, and 200 μg/mL	β-pinene, (Z)-β-ocimene, and α-pinene were the major compounds	72 h	no	The cytotoxic effect of EOs was prominent against HeLa, LS174, and A549 cell lines. EOs did not show toxicity side effects against normal MRC-5 cell (IC_50_ >200 μg/mL).
Wang et al. [95]	Murine macrophage RAW264.7 cells	*Trachydium roylei*	1.25, 2.5, 5.0, 10, and 20 mg/mL	β–phellandrene, myristicin, and elemicine were the major compounds	1 h	yes (100 ng/mL)	In LPS-stimulated RAW264.7 cells, only a high concentration of EOs (40 mg/mL) showed a negative effect on cell viability. In addition, incubation with EOs inhibited the production of TNF-α, IL-1β, and IL-6, whereas it increased the release of IL-10. EOs also inhibited the secretion of NO and PGE_2_.

Abbreviations: EO: essential oil; LPS: lipopolysaccharide; IL: interleukin; TNF-α: tumor necrosis factor alpha; NO: nitric oxide; iNOS: inducible nitric oxide synthase; MAPK: mitogen-activated protein kinase; PKB: protein kinase B; NF-κB: nuclear factor kappa-light-chain-enhancer of activated B cells; COX-2: cyclooxygenase 2; PGE2: prostaglandin E2; ox-LDL: oxidized low-density lipoprotein.

**Table 5:** Main characteristics of studies related with immunomodulatory properties of essential oils in animals.

Author	Animal	Plant Derived EOs	EOs Concentration		Main Components of EOs	EOs Administration	Treatment Duration	Main Results
Adaszynska-Skwirzynska et al. [96]	Broiler chickens	*Lavandula angustifolia*	0.4 mL/L	Linalool and linalool acetate were the major compounds (>80%)		Drinking water (6 h/day)	From 1 to 42 d of age and from 22 to 42 d of age	Broiler chickens treated with EO weighed an average of 6.35% more than those in the control group. No differences were found in feed and water intake, survival rate, or biochemical parameters. EOs intake also has an impact on ileum gastrointestinal microbiota (pathogenic microorganisms decreased, while the number of probiotic bacteria increased).
Altop et al. [97]	Broiler chickens	Liquidambar	0.0405, 0.0811, and 0.1622 g/kg	γ-Terpinen, terpinen-4-ol, and α-terpinene were the major compounds		Basal diet supplemented (ad libitum)	42 d	Treatment with EOs, mainly at 0.0811 g/kg concentration, improved growth performance and carcass traits while reducing blood cholesterol levels and *E. coli* counts.
Cetin et al. [98]	Broiler chickens	*Origanum* sp, *Rosmarinus officinalis* L and *Foeniculum vulgare* L.	Individual EO: 100 mg/kg. EO mixture: 100, 200 and 400 mg/kg	Rosemary oil, 1,8-cineol, α-pinene, and camphene; oregano oil, carvacrol; and fennel oil, trans-anethole, and fenchone		Basal diet supplemented (ad libitum)	42 d	Dietary supplementation increased the body weight of broilers at 7, 14, and 21 d of age. The blend of EO at 400 mg/kg significantly increased *Lactobacillus* spp. in feces, and also exhibited stronger antibacterial activity against coliform bacteria.
Chen et al [89]	C57BL/6 mouse	*Litsea cubea* L.	50- and 100-fold diluted	Terpene aldehydes (75.09%) were the most abundant compounds		Abdomens were painted	5 d	Treatment with EO showed an inhibitory effect on contact hypersensitivity response.
Chen et al. [22]	C57BL/6 mouse	*Artemisia argyi*	750, 250, and 83 mg/kg	Cineole, camphor, (−)-borneol, and α-(−)-thujone were the major compounds		Oral administration	30 minutes before 12-O-tetradeconoylphorbol-13-acetate application	Oral administration of the EO significantly attenuated TPA-induced mouse ear edema and decreased the protein level of COX-2
Gomes Cairo et al. [99]	Weaned pigs	*Schinus terebinthifolius Raddi*	0.5, 1.0, and 1.5 g/kg	𝛿-3-carene, 𝛼-phellandrene, limonene, and 𝛼-pinene were the major compounds		microencapsulated product	14 d	EO treatment modulated the gastrointestinal microbiota by increasing *Lactobacillus* and reducing enterobacteria counts. Growth performance was not affected by EO treatment, although EO (1.5 g/kg) can reduce diarrhea incidence.
Li et al. [100]	Weaned piglets	Carvacrol and thymol	Carvacol: 62.5 mg/kg; Thymol: 7.5 mg/kg	N-(2-hydroethyl)-iminodiacetic acid 2		Basal diet supplemented (ad libitum)	30 d	EO treatment significantly increased the relative abundance of *Bacillli*, *Lactobacillales*, *Streptocpccaceae* and *Veillonellaceae* in colonic samples. Metabolomics analysis showed that protein biosynthesis, amino acid metabolism, and lipid metabolisms were enriched in the EO group.
Park et al. [94]	Carrageenan-induced paw edema model (C57BL/6) and thioglycollate-induced peritonitis model (C57BL/6)	*Chamaecyparis obtusa*	5 and 10 mg/kg	α-terpinyl acetate, β-phellandrene, β-myrcene, limonene, bornyl acetate, γ-terpinene, β-thujaplicin, and α-terpineol		Intraperitoneal administration	1 h prior to inflammation-induced treatment	EO treatment reduced the levels of IL-6 and IL-1β in paw homogenates and in peritoneal fluid. In thioglycollate-induced peritonitis levels of TNF-α in peritoneal fluid.
Sutili et al. [101]	Silver catfish	*Hesperozygis ringes*, *Ocimun gratissimum*, and *Ocimun americanum*	*Hesperozygis ringes*: 20 and 40 mg/L; *Ocimun gratissimun:* 5 and 10 mg/L; *Ocimum americanun*: 10 and 20 mg/L	*H. ringens*, pulegone; *O. gratissimum*, eugenol; *O. americanum*, 1·8-cineole, β-linalool, eugenol, and camphor		Daily bath for	1 h during 5 d	Fish exposed to EOs showed significant lower hematocrit values and higher complement system activity and plasma cortisol levels. There was no significant difference in the survival of fish challenged with *Aeromonas hydrophila*.
Yang et al. [23]	Weaned piglets	Mixture of EOs and organic acids: cinnamaldehyde (15%), thymol (5%), citric acid (10%), sorbic acid (10%), malic acid (6.5%) and fumaric acid (13.5%)	1 kg/ton			Basal diet supplemented (ad libitum)	28 d	Diet supplementation with the mixture improved the final body weight and average daily gain, increased the concentration of serum complement 4, and enhanced the isovaleric acid fecal concentration. Regarding the gastrointestinal microbiota composition in fecal samples, the mixture treatment increased the beta diversity.

Abbreviations: EO, essential oil; d: days; TNF-α: tumor necrosis factor alpha; TPA: 12-O-tetradeconoylphorbol-13-acetate; COX-2: ciclooxigenase 2.
